# Clinical Outcomes of a Novel Unidirectional Porous β-Tricalcium Phosphate Filling in Distal Radius Fracture with Volar Locking Plate Fixation: Secondary Publication of the Japanese Version

**DOI:** 10.3390/medicina60010001

**Published:** 2023-12-19

**Authors:** Yoshito Sudo, Yoshihiro Nishida, Hiroatsu Nakashima, Tetsuya Arai, Tetsuro Takatsu

**Affiliations:** 1 Department of Orthopaedic Surgery, Gifu Prefectural Tajimi Hospital, Gifu 507-8522, Japan; sudo.yoshito.106@gmail.com (Y.S.); g88o7s2n@sf.commufa.jp (H.N.); abeetle2009@yahoo.co.jp (T.A.); te2tkt@gmail.com (T.T.); 2 Department of Rehabilitation Medicine, Nagoya University Hospital, Nagoya 466-8550, Japan; 3 Medical Corporation Rokujukai, Goto Orthopaedic Clinic, Tsuhima 496-0072, Japan

**Keywords:** radius, fracture, unidirectional porous β-tricalcium phosphate, bone graft, surgery

## Abstract

Postoperative loss of correction is a concern in cases of distal radius fracture with bone loss after surgery. The purpose of this study was to evaluate the usefulness of a β-tricalcium phosphate (β-TCP) with unidirectional pore structure (Affinos^®^: Kuraray Co., Ltd, Tokyo, Japan) with internal fixation in patients with bone defects during the correction of distal radius fractures. Thirty-nine patients (40 radii) treated between 2016 and August 2020 were included in the study. There were 8 males and 31 females; the mean age was 70.9 (32–88). The mean postoperative observation period was 14.6 (3.4–24) months. The bone defect that occurred in the surgery was filled with Affinos^®^ and fixed with a locking plate. Radial inclination (RI), volar tilt (VT), and ulnar variance (UV) were evaluated after the operation and at the final observation. The start of absorption and the completion of replacement to the host bone of Affinos^®^ were also evaluated. There were no complications associated with grafts of Affinos^®^. The mean time of translucent findings around artificial bone was 1.85 (0.5–6) months, and that of complete resorption was 10.6 (1.5–16.5) months after surgery. The mean RI was 21.82° after surgery and 21.16° at final observation. The mean VT was 8.54° after surgery and 8.50° at final observation. The mean UV was −0.3 mm after surgery and 0.5 mm at final observation. Affinos^®^ was resorbed relatively early, and host bone formation was observed. Filling of unidirectional pore structure β-TCP with internal fixation showed favorable outcomes in the surgery of distal radius fractures with bone defects.

## 1. Introduction

According to the World Health Organization, distal radius fractures are the most common osteoporotic fractures in older women and are one of the top 10 most costly medical accidents in the world [1].

To avoid potentially serious complications such as post-traumatic osteoarthritis and joint contractures, the treatment of distal radius fractures requires that the healing process stabilizes the fracture site and properly aligns the fracture site. The volar approach and locking plate fixation are currently widely used as open reduction and internal fixation. With this method, both anatomically appropriate fracture reduction and better clinical and functional outcomes could be obtained [2].

The addition of bone grafting in the surgical treatment of distal radius fractures remains controversial. Traditionally, the standard of choice for bone grafting has been autologous bone grafting, typified by autologous iliac bone grafting. However, this technique has several limitations, including the need for additional surgical approaches to the donor site and complications [3]. Allogeneic bone grafting is an option; however, it may be difficult to obtain in some countries, particularly in Japan. Compared to these bone graft materials, artificial bone grafts have become increasingly popular among alternative strategies because they are commercially available and easy to adapt to the graft site.

In the case of distal radius fractures under the condition of osteoporotic or comminuted fractures, filling the bone defect with artificial bone is reported to maintain the correct position and promote bone fusion [4].

As described, autologous bone grafting may be the best choice. However, it has the drawback of invading the donor site. Although correction loss is one of the major concerns after distal radius fracture, there are few studies reporting correction loss after artificial bone filling after surgery.

In our hospital, when performing surgery for a distal radius fracture with a bone defect after correction, β-TCP with an oriented continuous porous structure, Kuraray’s Affinos**^®^** has been used as filling and fixed with a locking plate. We hypothesized that this novel artificial bone could reduce the correction loss after surgery and improve the bone union in patients with distal radius fractures, particularly in unstable and elderly cases. In the present study, clinical outcomes of grafted artificial bone for distal radius fracture surgery were investigated, focusing on the filling resorption and replacement to host bone, in addition to correction loss during follow-up in patients treated with internal fixation and Affinos**^®^**.

## 2. Materials and Methods

This is a retrospective case series performed in Gifu Prefectural Tajimi Hospital. From January 2016 to August 2020, forty distal radius fractures (39 patients) were treated at our hospital. There were 8 males and 31 females. The mean age was 70.9 years (32–88 years). Patients with minimal bone defect or without osteoporosis were excluded from the present study because it seemed that artificial bone grafting was not necessary. According to the AO classification, there were A3 with 4, B1 with 2, B3 with 3, C1 with 10, C2 with 13, and C3 with 8. The mean postoperative observation period was 14.6 months (3.4–24 months). All patients received surgical treatment under anesthesia with axillary block without general anesthesia and sedation. The incision was made along the palpable flexor carpi radialis (FCR) tendon sheath (Trans-FCR approach). The pronator quadratus muscle was dissected near the radius and inverted, and the fracture site was expanded. The fractured part was reduced, and the resulting bone defect was filled with a B01 Affinos^®^ (a cube of 1 cm per side). If the block was too large for the bone defect area, it was molded with a ruel, etc. After the status of the fracture reduction and artificial bone filling were confirmed to be adequate under direct vision and/or fluoroscopy, the fracture site was temporally fixed with Kirschner steel wire. Then, the locking plate was placed on the volar cortex and fixed by locking screws. Postoperatively, the patient was immobilized externally for 2 weeks generally, and then rehabilitation, including range of motion training, was started. With X-ray, the following factors were evaluated at 2 weeks, 1 month, 2 months, 3 months, 6 months, and 9 months postoperatively: radial inclination (RI), volar tilt (VT), and ulnar variance (UV) immediately after surgery and at the time of final observation. Resorption and replacement to the host bone of the grafted artificial bone. An evaluator was blinded to clinical data (Y.S.). The range of motion of the wrist joint and grip strength were also evaluated at the final observation. This study was approved by the institutional review board of Gifu Prefectural Tajimi Hospital (approved number: 2022-20-1). In this approval, the need for informed consent was waived because of the retrospective design of the study based on anonymous data.

Statistical analysis. The mean value was used to express age, observation period, RI, VT, UV, range of motion, and grip strength.

## 3. Results

The RI averaged 21.82° immediately after surgery and 21.16° at the last observation. The correction loss averaged 0.67°. VT averaged 8.54° immediately after surgery and 8.50° at the last observation, with an average correction loss of 0.04°. UV averaged −0.33 mm immediately after surgery and 0.53 mm at the last observation, with a mean correction loss of 0.85 mm (Table 1). All three cases with a large correction loss of RI (>5°) were C2 according to the AO classification. One case with a large correction loss of VT (>5°) was A3. The mean time of resorption (appearance of translucent image) of grafted artificial bone was 1.85 months (0.5–6), and the mean time of complete replacement (disappearance) to the host bone was 10.6 months (1.5–16.5). At the time of the final follow-up, the mean palmar and dorsal flexion of the wrist joint was 64.8°and 60.8°, and the grip strength was 86.6% of the healthy side. No related complications were observed due to the use of β-TCP.

### Case Presentation

**Figure 1:**
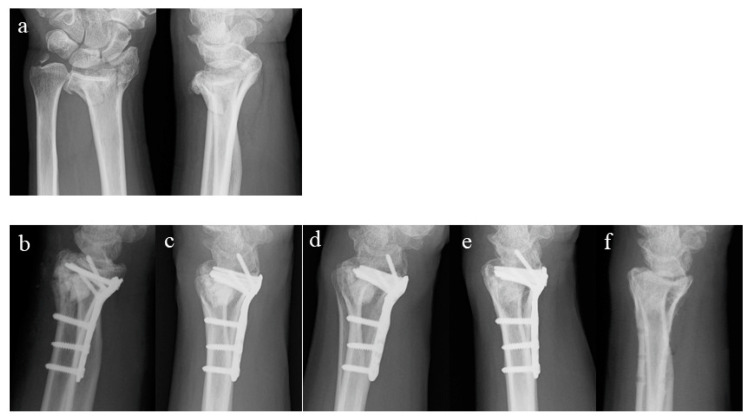
A 63-year-old man. Left distal radius fracture, AO classification C2. (**a**) Preoperative (antero-posterior, lateral); (**b**) immediate, postoperative; (**c**) 3 months (absorption of artificial bone in the periphery); (**d**) 6 months; (**e**) 12 months; (**f**) 15 months (artificial bone complete replacement to host bone).

In this case, we studied a 63-year-old male. He fell from a chair and was injured. The fracture type was AO classification C2. Three months after the surgery, a translucent area appeared in the periphery of Affinos^®^, and it was completely absorbed at the time of 15 months. Corrective loss was 1.63° for RI, 0.71° for VT, and 1 mm for UV, and the reduction status of the fracture site was maintained (Figure 1).

## 4. Discussion

The results in the present study showed that correction loss of RI, VT, and UV using a novel artificial bone with unidirectional porous structure were 0.67°, 0.04°, and 0.85 mm, respectively. These values appear to be satisfactory. Kuraray’s Affinos^®^ is invented as tricalcium beta-phosphate with an oriented interconnected porous structure. It has a porosity of 57% and unidirectional pores promoting good entry and growth of blood vessels and bone inside. Affinos has been developed in cube, cylinder, and granule shapes, with a strength of 14.4 MPa, which is reported to be comparable to cancellous bone [5]. There are few reports of clinical studies using Affinos^®^ for fractures. Izawa et al. [6] used Affinos^®^ in 4 cases of calcaneal fractures and reported that its resorption started at 3 months after surgery and replaced to host bone at 6 months in the earliest case. Funayama et al. [7] used Afinos^®^ in 5 cases of burst vertebral fractures, showing that it was replaced to host bone at 3 months postoperatively in one case, and in other 4 cases, the resorption started at 3 months postoperatively and completed at 12 months. It suggested that Affinos has good properties of material resorption and replacement to host bone. Regarding fractures of the distal radius, Muramatsu et al. [8] only reported the clinical outcomes of Affinos^®^ filling in the surgical treatment. The average age in their study was 74 years, and in the present study, it was almost the same as 71 years. The number of patients was 36 in their study, which was almost the same as 39 (40 radii) in the present study. The results of their study indicated that resorption of artificial bone began 2 months after surgery, and replacement to host bone occurred at 5 months. In the present study, resorption started early in the postoperative period, and the bone was replaced in less than a year, which was consistent with the results of Muramatsu’s study. However, the present study is the first to investigate all cases of correction loss, indicating that Affinos filling may reduce correction loss in distal radius fractures in the elderly.

The effects of bone-filling materials on distal radius fractures, particularly the need and effects of bone-filling materials when using internal fixation, have been poorly reported, unclear, and controversial. Goto et al. [9] reported that using hydroxyapatite with a palmar locking plate, the filled group had a significantly less corrective loss of UV. Okano et al. indicated that using an oriented continuous porous hydroxyapatite with a palmar locking plate, there was a tendency for less corrective loss of VT in the filled group. Chang et al. [1] noted that the use of a hydroxyapatite compound with a palmar locking plate had no significant effects on radiological correction loss. In the present study, the cohort was composed of most AO classification C-type patients, indicating a high degree of comminution and instability. However, the correction loss of VT and UV was minimum, which suggests the effectiveness of Affinos^®^ filling. On the other hand, it is necessary to compare the clinical results of osteosynthesis using a locking plate without using artificial bone with the clinical results of this study. There is a report on radiological evaluation of female patients over 50 years old, divided into groups with osteoporosis (group 1) and groups without osteoporosis (group 2) [10]. The mean correction loss of RI was 0.80°, and that of VT was 2.13° in group 2. The correction loss of RI and VT was superior in the present study, suggesting the effectiveness of Affinos^®^ filling. There were two randomized controlled trials (RCTs) investigating the utility of volar plate fixation compared with external fixation. The results of one RCT revealed that external fixation did not maintain radial length to the same extent as volar plates did. However, both groups lost reduction in volar tilt over time; 5° in the volar plates group at 1 year after surgery [11]. The results of the other RCT [12] indicated that the volar locking plate led to better patient-reported outcomes compared with external fixation or radial column plate, whereas the mean correction loss of RI was 3.5° and that of VT was 0.1° between 6 and 12 months after surgery in the group of volar locking plates, which was inferior to those in the present study.

The present study has limitations. The first is that only patients with Affinos^®^ were studied, and comparisons with unused patients were not performed. The second is that there is only one observer to evaluate the absorption time of Affinos^®^. The third, functional, and patient-reported outcome was not evaluated.

## 5. Conclusions

Oriented porous β-TCP filling with internal fixation for distal radius fracture with bone loss may be promising to prevent correction loss such as dorsiflexion dislocation and shortening.

## Figures and Tables

**Table 1:** Mean RI, VT, UV, and mean corrective loss.

	Pre-Ope	Final Observation	Correction Loss
RI	21.82°	21.16°	0.67°
VT	8.54°	8.50°	0.04°
UV	−0.33 mm	+0.53 mm	0.85 mm

## Data Availability

Data used to support the findings of this study are available from the corresponding author upon request.
